# Editorial: Genetically engineered products: Preparing for the future

**DOI:** 10.3389/fbioe.2022.1077237

**Published:** 2022-11-22

**Authors:** Patricia Machado Bueno Fernandes, Carmen Vicien, Deise Maria Fontana Capalbo

**Affiliations:** ^1^ Biotechnology Core, Federal University of Espírito Santo, Vitória, Brazil; ^2^ Faculty of Agronomy, University of Buenos Aires and Institute for Scientific Cooperation in Environment and Health, Buenos Aires, Argentina; ^3^ Brazilian Agricultural Research Corporation (EMBRAPA), Brasília, Brazil

**Keywords:** GM regulation, public perception, risk management, CRISPR, bioengineering

The benefits of first-generation GM crops are remarkable and could be greater if there had been wider adoption of these technologies. Looking back, many of the constraints, discussions, and difficulties observed in the registration and commercialization of GM crops were based on regulatory structures and risk analysis topics. On one side, the regulatory structure varied from country to country and on the other side, the varied requirements made it difficult to have GM products approved. Due to this, only private companies were economically prepared to reach the market.

However, new technologies such as gene editing proved to be more specific, faster, and predictable, and, mainly, had lower regulatory costs. Therefore, they can be developed for the market by small companies and research institutes and may contribute to major environmental policy initiatives as many products under development in plants, animals, and microorganisms are designed to provide specific environmental benefits. Nevertheless, this would require researchers and developers of gene-edited products to have a clearer understanding of the regulatory landscape and how a product moves from early development to commercialization.

All this leads to the main objective of this Research Topic, which seeks to undertake a brief retrospective examination of the positive and negative effects of GM materials, mostly considering the relationship between regulation and innovation, with specific attention to gene editing techniques. Aspects related to public perception and communication were also taken into account. This would allow us to envisage the future.

With a retrospective look at 30 years of regulatory submission data, George et al. try to understand and forecast how the new SECURE rule from APHIS in the US might affect future diversification trends. In a more recent case, Vesprini et al. present some important modifications enacted during 2020 and 2021 in Argentina’s regulatory policies on the Environmental Risk Assessment (ERA), thus exploring the possibilities of introducing novel approaches to enhance the ERA and make it more efficient by applying scientific criteria and the accumulated experience and scientific bibliography on the Research Topic. Rocha-Salavarrieta also brings an important case from Latin American countries, related to regulatory harmonization. This harmonization is, in itself, a government responsibility, since governments define, implement, and are responsible for what and how to regulate. Harmonization can be aided by aligning definitions, standardizing the information needed to make informed decisions, defining timeframes for making determinations, and contemplating the possible recognition of decisions made by other countries. This article describes how those Research Topics can be addressed in a cooperative way, by neighboring countries, to effectively contribute to safe biotechnology development.

Gene-edited products bring an opportunity for the creative adaptation of the current regulatory regimes, to learn from the experience of the safe use of GM technologies, and allow for the opening of innovation opportunities beyond the limited range of basic crops. In a review of CRISPR/CAS- and topical RNAi-based technologies for crop management and improvement, Távora et al. address several aspects related to risk assessment, toxicity, and advances in the use of these tools. For Argentina’s regulatory system, Goberna et al. examine how regulatory management took advantage of scientific progress to boost innovation and give more opportunities to local developers. Dealing with the uncertainties and risks of new genomic techniques, another publication, from Bouchaut et al., shows results from five workshops based on one case (genetic engineering of plants’ rhizosphere) trying to identify tensions between different stakeholder groups. The authors propose a tool—a script on how to organize a stakeholder workshop—using anticipatory strategies to lower or mitigate uncertainties, helping to identify knowledge gaps as well. Jordan et al. report the findings from interviews and deliberative workshops from a broad multi-sector deliberative group and consider the merits of gene editing relative to alternative plant-breeding methods as a means for improving crops for Continuous Living Cover (CLC) agriculture, which they consider a powerful tool for developing and expanding to scale. In this sense, Fernandes et al. discuss how the long-overdue partnership between biotechnology and organic agriculture is fundamental for the mitigation of food insecurity and is a way forward to truly sustainable agriculture. They point out that if regulatory hurdles are not unfeasible, CRISPR technology and its derived seeds will be viable for small family farmers and could be the basis of sustainable organic agriculture.

Another Research Topic is that of consumer concerns; being well known that public opinion is ambivalent or critical towards foods derived from GM materials. Therefore, Collazo et al., address attitudes of the Ecuadorian University Community toward GM organisms based on socio-demographic variables, knowledge, beliefs, practices, and bioethical approach, indicating an incipient acceptance of GM organisms in the academic sector that might corroborate a transformation in the thinking of Ecuadorian civil society.

More traditional aspects of the environmental effects of GM products are reviewed and analyzed in order to discuss what and how new technologies could benefit their risk-benefit balance, using previous GM studies. Seixas et al. review and discuss the environmental effects due to pesticides for two different GM seeds, insect-resistant cotton and herbicide-tolerant soybeans, in a particular period of Brazilian agriculture from 2009–2013, using a dataset on commercial farms’ use of pesticides and biotechnology. Horizontal gene transfer (HGT), i.e., the acquisition of genetic material that has not been inherited from a parent, assessments utilizing new tools for detection as well as next-generation sequencing are presented by Philips et al. Their discussion leads to an updated view of the likelihood, factors, and barriers to the occurrence of HGT in a variety of recipients, using mainly the framework of the Australian legislation.

As bioengineering advances, Gemler et al. describe the need for a biohazard review, shifting from organism-based analyzes to function-centered classifications. They present a new methodology for classifying biohazards at the individual sequence level, which they have compiled to distinguish the biohazard property of pathogenicity at the whole genome level. The resulting database can be used to develop hazardous “fingerprints” based on the functional metadata categories. The authors foresee that such a shift could lead to the improvement and standardization of current biosecurity and biosafety practices.

In conclusion, this Research Topic provided a multidisciplinary view of GMO regulation, focusing on relevant aspects of politics, economics, agronomics, health, and the safety of GE products. It covers a wide range of articles and reviews within the field, grouping a series of results with impacts and potential benefits of GE products to society, food/feed chains, and the environment.

